# Information Entropy Production of Maximum Entropy Markov Chains from Spike Trains

**DOI:** 10.3390/e20010034

**Published:** 2018-01-09

**Authors:** Rodrigo Cofré, Cesar Maldonado

**Affiliations:** 1 Centro de Investigación y Modelamiento de Fenómenos Aleatorios, Facultad de Ingeniería, Universidad de Valparaíso, Valparaíso 2340000, Chile; 2 IPICYT/División de Matemáticas Aplicadas, Instituto Potosino de Investigación Científica y Tecnológica, San Luis Potosí 78216, Mexico

**Keywords:** information entropy production, discrete Markov chains, spike train statistics, Gibbs measures, maximum entropy principle

## Abstract

The spiking activity of neuronal networks follows laws that are not time-reversal symmetric; the notion of pre-synaptic and post-synaptic neurons, stimulus correlations and noise correlations have a clear time order. Therefore, a biologically realistic statistical model for the spiking activity should be able to capture some degree of time irreversibility. We use the thermodynamic formalism to build a framework in the context maximum entropy models to quantify the degree of time irreversibility, providing an explicit formula for the information entropy production of the inferred maximum entropy Markov chain. We provide examples to illustrate our results and discuss the importance of time irreversibility for modeling the spike train statistics.

## 1. Introduction

In recent years, multi-electrode arrays and neuroimaging recording techniques have allowed researchers to record simultaneously from large populations of neurons [1]. Analysis carried on the recorded data has shown that the neuronal activity is highly variable (even when presented repeatedly the same stimulus). The observed variability is due to the fact that noise is ubiquitous in the nervous system at all scales, from ion channels through synapses up to the system level [2,3,4]. The nature of noise in the nervous system thus determines how information is encoded [5,6,7]. In spite of the different sources of noise, the spiking response is highly structured in statistical terms [8,9,10], for that reason many researchers have hypothesized that the population neural code is largely driven by correlations [10,11,12,13,14,15].

There are numerous sources of spike correlations that involve time delays, such as the activity of an upstream neuron projecting to a set of the observed neurons [16], top-down delayed firing rate modulation [17], among others. As discussed in [18], spike interactions in different times could have a non-negligible role in the spike train statistics. Indeed, there is strong evidence that interneuron temporal correlations play a major role in spike train statistics [19,20,21,22].

Since spikes are stereotyped events, the information about spikes is conveyed only by its times of occurrence. Considering small windows of time for each neuron, either a spike occurs in a given interval or not, producing in this way binary sequences of data easier to analyze statistically. However, traditional methods of statistical inference are useless to capture the collective activity under this scenario since the number of possible spike patterns that a neural network can take grows exponentially with the size of the population. Even long experimental recordings usually contain a very small subset of the entire state space, which makes the empirical frequencies poor estimators for the underlying probability distribution.

Since the spiking data are binary, it is natural to attempt to establish a link between neural activity and models of spins over lattices from statistical mechanics. Since the seminal work of Jaynes [23] a succession of research efforts have helped to develop a framework to characterize the statistics of spike trains using tools the maximum entropy principle (MEP). This approach is promising since the MEP provides a unique statistical model for the whole spiking neuronal network that is consistent with the average values of certain features of the data but makes no additional assumptions. In Schneidman et al. [10] and Pillow et al. [24], the authors used the maximum entropy principle focusing on firing rates and instantaneous pairwise interactions (Ising model) to describe the spike train statistics of the vertebrate retina responding to natural stimuli. Since then, the MEP approach has become a standard tool to build probability measures in this field [10,21,24,25]. Recently, several extensions of the Ising model have been proposed, for example, the triplet model, considering as an extra constraint, the correlation of three neurons firing at the same time [15], and the so-called *K*-pairwise model which consider *K* neurons firing at the same time bin [25]. These studies have raised interesting questions about important aspects of the neuronal code such as criticality, redundancy and metastability [25,26].

Although relatively successful in this field, this attempt of linking neural populations and statistical mechanics is based on assumptions that go against fundamental biological knowledge. In particular, most of these works have focused only on synchronous constraints and thus, modeling time-independent processes which are reversible in time. From a fundamental perspective, since a population of neurons is a living system it is natural to expect them not to be characterized by i.i.d. random variables. As such, the statistical description of spike trains of living neuronal networks should reflect irreversibility in time [27], and hence require a description based on out-of-equilibrium statistical mechanics. Thus, quantifying the degree of time irreversibility of spike trains becomes an important challenge, which, as we show here, can be approached using tools from the fruitful intersection between information theory and statistical mechanics. Given a stochastic system, the quantity that measures how far it is from its equilibrium state (in statistical terms) is called *information entropy production* (IEP) [28] (We distinguish the information entropy production with others forms of entropy production used in chemistry and physics).

The maximum entropy approach can be extended to include non-synchronous constraints within the framework of the thermodynamic formalism and Gibbs measures in the sense of Bowen [29] (the notion of the Gibbs measure extends also to processes with infinite memory [30], and have been used in the context of spike train statistics [31,32]). This opens the possibility to capture the irreversible character of the underlying biological process, and, thus, build more realistic statistical models. In this paper, we quantify the IEP of maximum entropy measures of populations of spiking neurons under arbitrary constraints and show that non-equilibrium steady states (NESS) emerge naturally in spike train statistics obtained from the MEP.

There is a vast body of theoretical work about the irreversibility of stochastic processes, for mathematical details we refer the reader to [28]. In particular, for discrete time Markov chains, Gaspard [33] deduced an explicit expression for the change in entropy as the sum of a quantity called entropy flow plus the entropy production rate. In this paper, we follow this expression adapted to Markov chains obtained from the MEP and we provide an explicit expression for the IEP of maximum entropy Markov chains (MEMC).

This paper is organized as follows: In Section 2, we introduce the setup of discrete homogeneous Markov chains and review the properties that we use further. In Section 3, we introduce the MEP within the framework of the thermodynamic formalism and Gibbs measures, discussing the role of the arbitrary constraints. We also provide the explicit formula to compute the IEP solely based on the spectral properties of the transfer matrix. In Section 4, we provide examples of relevance in the context of spike train statistics. We finish this paper with discussions pointing out directions for further research.

## 2. Generalities

To set a common ground for the analysis of the IEP of spike trains, which are time-series of action potentials (nerve impulses) emitted by neurons, these spikes are used to communicate with other neurons. Here, we introduce the notations and provide the basic definitions used throughout the paper.

### 2.1. Notation

We consider a finite network of N≥2 neurons. Let us assume that there is a natural time discretization such that at every time step, each neuron emits at most one spike (There is a minimal amount of time called “refractory period” in which no two spikes can occur. When binning, one could go beyond the refractory period and two spikes may occur in the same time bin. In those cases, the convention is to consider only one spike). We denote the *spiking-state* of each neuron $\sigma_{k}^{n}=1$ whenever the *k*-th neuron emits a spike at time *n*, and $\sigma_{k}^{n}=0$ otherwise. The spike-state of the entire network at time *n* is denoted by $\sigma^{n}:={\left[\sigma_{k}^{n}\right]}_{k=1}^{N}$, which we call a *spiking pattern*. For $n_{1}\leq n_{2}$, we denote by $\sigma^{n_{1},n_{2}}$ to an ordered concatenation of spike patterns

$$\sigma^{n_{1},n_{2}}=\sigma^{n_{1}}\sigma^{n_{1}+1}\cdots \sigma^{n_{2}-1}\sigma^{n_{2}},$$

that we call *spike block*. We call the sample of *T* spiking patterns a *spike train*, which is a spike block $\sigma^{0,T}$. We consider also infinite sequences of spike patterns that we denote $\bar{\sigma }$. We denote the set of infinite binary sequences of *N* neurons $\Sigma_{N}$.

Let L>0 be an integer, we write $\Sigma_{N}^{L}={\left\{0,1\right\}}^{N\times L}$ for the set of spike blocks of *N* neurons and length *L*. This is the set of N×L blocks whose entries are 0’s and 1’s. We introduce a symbolic representation to describe the spike blocks. Consider a fixed *N*, then to each spike block $\sigma^{0,L-1}$ we associate a unique number ℓ∈N, called *block index*:

(1) $$\ell =\sum_{k=1}^{N}\sum_{n=0}^{L-1}2^{n\;N+k-1}\;\sigma_{k}^{n}.$$

We adopt the following convention: neurons are arranged from bottom to top and time runs from left to right in the spike train. For fixed *N* and *L*, $\sigma^{\left(\ell \right)}$ is the unique spike block corresponding to the index *ℓ*.

### 2.2. Discrete-Time Markov Chains and Spike Train Statistics

Let $\Sigma_{N}^{L}$ be the state space of a discrete time Markov chain, and let us for the moment use the following notation $\sigma_{\left(n\right)}:=\sigma^{n,n+L-1}$, for the random blocks and analogously $\omega_{\left(n\right)}:=\omega^{n,n+L-1}$ for the states. Consider the process $\left\{\sigma_{\left(n\right)}:n\geq 0\right\}$. If $\sigma_{\left(n\right)}=\omega_{\left(n\right)}$, we say that the process is in the state $\omega_{\left(n\right)}$ at time *n*. The transition probabilities are given as follows,

(2) $$P\left[\sigma_{\left(n\right)}=\omega_{\left(n\right)}|\sigma_{\left(n-1\right)}=\omega_{\left(n-1\right)},\cdots ,\sigma_{\left(0\right)}=\omega_{\left(0\right)}\right]=P\left[\sigma_{\left(n\right)}=\omega_{\left(n\right)}|\sigma_{\left(n-1\right)}=\omega_{\left(n-1\right)}\right].$$

We assume that this Markov chain is homogeneous, that is, (2) is independent of *n*. Consider two spike blocks $\sigma^{0,L-1},{\tilde{\sigma }}^{1,L}\in \Sigma_{N}^{L}$ of length L≥2. Then, the transition $\sigma_{\left(0\right)}\to {\tilde{\sigma }}_{\left(1\right)}$ is *allowed* if they have the common sub-block $\sigma^{1,L-1}={\tilde{\sigma }}^{1,L-1}$.

We consider Markov transition matrices $P:\Sigma_{N}^{L}\times \Sigma_{N}^{L}\to R$, whose entries are given by:

(3) Pσ(0),σ˜(1):=P[σ˜(1)∣σ(0)]>0if σ(0)→σ˜(1) isallowed0,otherwise.

Note that *P* has $2^{NL}\times 2^{NL}$ entries, but it is a sparse matrix since each row has, at most, $2^{N}$ non-zero entries. Observe that by construction, for any pair of states there is a path of maximum length *L* in the graph of transition probabilities going from one state to the other, therefore the Markov chain is irreducible.

### 2.3. Detailed Balance Equations

Consider a fix *N* and *L*. From the Markov property and the definition of the homogeneous transition matrix, one has for an initial measure ν, the following Markov measure μ(ν,P)

(4) $$\mu \left[\sigma_{\left(0\right)}=\omega_{\left(0\right)},\sigma_{\left(1\right)}=\omega_{\left(1\right)},\cdots ,\sigma_{\left(k\right)}=\omega_{\left(k\right)}\right]=\nu \left(\omega_{\left(0\right)}\right)P_{\omega_{\left(0\right)},\omega_{\left(1\right)}}\cdots P_{\omega_{\left(k-1\right)},\omega_{\left(k\right)}},$$

for all k>0. Here, again, we used the short-hand notation $\sigma_{\left(k\right)}:=\sigma^{k,L+k-1}$ and $\omega_{\left(k\right)}:=\omega^{k,L+k-1}$.

An *invariant probability measure* of a Markov transition matrix *P* is a row vector π such that

(5) πP=π.

We recall that, for ergodic Markov chains (irreducible, aperiodic and positive recurrent), the invariant measure is unique.

Let us now consider a more general setting including non-stationary Markov chains. Let $\nu^{n}$ be the distribution of blocks $\sigma^{\left(\ell \right)}\in \Sigma_{N}^{L}$ at time *n*, then one has that the probability evolves in time as follows,

$$\nu^{n+1}\left(\sigma^{\left(\ell \right)}\right)=\sum_{\sigma^{\left(\ell^{\prime }\right)}\in \Sigma_{N}^{L}}\nu^{n}\left(\sigma^{\left(\ell^{\prime }\right)}\right)P_{\ell^{\prime },\ell }.$$

For every $\sigma^{\left(\ell \right)}\in \Sigma_{N}^{L}$, one may write the following relation

(6) $$\nu^{n+1}\left(\sigma^{\left(\ell \right)}\right)-\nu^{n}\left(\sigma^{\left(\ell \right)}\right)=\sum_{\sigma^{\left(\ell^{\prime }\right)}\in \Sigma_{N}^{L}}\left[\nu^{n}\left(\sigma^{\left(\ell^{\prime }\right)}\right)P_{\ell^{\prime },\ell }-\nu^{n}\left(\sigma^{\left(\ell \right)}\right)P_{\ell ,\ell^{\prime }}\right].$$

This last equation is related to the conditions of reversibility of a Markov chain. When stationarity and ergodicity are assumed, the unique stationary measure of the Markov chain π is said to satisfy detailed balance if:

(7) $$\pi_{\ell }P_{\ell ,\ell^{\prime }}=\pi_{\ell^{\prime }}P_{\ell^{\prime },\ell }\;\forall \sigma^{\left(\ell \right)},\sigma^{\left(\ell^{\prime }\right)}\in \Sigma_{N}^{L}.$$

If the detailed balance equations are satisfied, then the quantity inside the parenthesis in the right-hand side of Equation (6) is zero.

### 2.4. Information Entropy Rate and Information Entropy Production

A well established measure of the amount of uncertainty of a probability measure ν is the *information entropy rate*, which we denote by S(ν). In the case of independent sequences of spike patterns (L=1), the entropy rate is given by:

(8) $$S\left(\nu \right)=-\sum_{\sigma^{\left(\ell \right)}\in \Sigma_{N}^{1}}\nu [\sigma^{\left(\ell \right)}]\log\nu [\sigma^{\left(\ell \right)}].$$

In the setting of ergodic stationary Markov chains taking values in the state space $\Sigma_{N}^{L};L\geq 2$ with transition matrix *P* and unique invariant measure π, the information entropy rate associated to the Markov measure μ(π,P) is given by:

(9) $$S\left(\mu \right)=-\sum_{\sigma^{\left(\ell \right)},\sigma^{\left(\ell^{\prime }\right)}\in \Sigma_{N}^{L}}\pi_{\ell }P_{\ell ,\ell^{\prime }}\log P_{\ell ,\ell^{\prime }},\;L\geq 2,$$

which corresponds to the *Kolmogorov–Sinai entropy* (KSE) [34].

Here, we introduce the information entropy production as in [33]. For expository reasons, let us consider again the non-stationary situation. The information entropy of a probability measure ν in the state space $\Sigma_{N}^{L}$ at time *n* be given by

$$S_{n}\left(\nu \right)=-\sum_{\sigma^{\left(\ell \right)}\in \Sigma_{N}^{L}}\nu^{n}\left(\sigma^{\left(\ell \right)}\right)\log\nu^{n}\left(\sigma^{\left(\ell \right)}\right).$$

The *change of entropy rate* over one time-step is defined as follows:

$$\Delta S_{n}:=S_{n+1}\left(\nu \right)-S_{n}\left(\nu \right)=-\sum_{\sigma^{\left(\ell \right)}\in \Sigma_{N}^{L}}\nu^{n+1}\left(\sigma^{\left(\ell \right)}\right)\log\nu^{n+1}\left(\sigma^{\left(\ell \right)}\right)+\sum_{\sigma^{\left(\ell \right)}\in \Sigma_{N}^{L}}\nu^{n}\left(\sigma^{\left(\ell \right)}\right)\log\nu^{n}\left(\sigma^{\left(\ell \right)}\right).$$

Rearranging the terms, one has that the previous equation can be written as:

(10) $$\begin{array}{cc} \Delta S_{n}= & \;-\sum_{\sigma^{\left(\ell \right)},\sigma^{\left(\ell^{\prime }\right)}\in \Sigma_{N}^{L}}\nu^{n}\left(\sigma^{\left(\ell^{\prime }\right)}\right)P_{\ell^{\prime },\ell }\log\frac{\nu^{n+1}\left(\sigma^{\left(\ell^{\prime }\right)}\right)P_{\ell^{\prime },\ell }}{\nu^{n}\left(\sigma^{\left(\ell \right)}\right)P_{\ell ,\ell^{\prime }}}+\;\\ & \;\frac{1}{2}\sum_{\sigma^{\left(\ell \right)},\sigma^{\left(\ell^{\prime }\right)}\in \Sigma_{N}^{L}}\left[\nu^{n}\left(\sigma^{\left(\ell^{\prime }\right)}\right)P_{\ell^{\prime },\ell }-\nu^{n}\left(\sigma^{\left(\ell \right)}\right)P_{\ell ,\ell^{\prime }}\right]\log\frac{\nu^{n}\left(\sigma^{\left(\ell^{\prime }\right)}\right)P_{\ell^{\prime },\ell }}{\nu^{n}\left(\sigma^{\left(\ell \right)}\right)P_{\ell ,\ell^{\prime }}}, \end{array}$$

where the first part on the r.h.s of this equation is called *information entropy flow* and the second *information entropy production* [33].

Observe that, in the stationary state, one has that $\nu^{n}=\nu^{n+1}=\pi$, thus the change of entropy rate is zero, meaning that information entropy flow equal information entropy production, therefore is possible to attain a steady state of fixed maximum entropy, but having positive IEP. In this case, we refer to NESS [35].

Here, since we are interested in the Markov chains that arise from the maximum entropy principle, we focus on the stationary case. In this case, the IEP of a Markov measure μ(π,P) is explicitly given by:

(11) $$IEP\left(P,\pi \right)=\frac{1}{2}\sum_{\sigma^{\left(\ell \right)},\sigma^{\left(\ell^{\prime }\right)}\in \Sigma_{N}^{L}}\left[\pi_{\ell^{\prime }}P_{\ell^{\prime },\ell }-\pi_{\ell }P_{\ell ,\ell^{\prime }}\right]\log\frac{\pi_{\ell^{\prime }}P_{\ell^{\prime },\ell }}{\pi_{\ell }P_{\ell ,\ell^{\prime }}}\geq 0,$$

nevertheless, we stress the fact that one can obtain the information entropy production rate also in the non-stationary case.

## 3. Maximum Entropy Markov Chains

Usually, one only have access to a limited amount of experimental spiking data, which is a sampling of a very small subset of the entire state space. This makes that often the empirical frequencies are bad estimations of the elements of the Markov transition matrix. Here, we present how to use a variational principle from the thermodynamic formalism [36] to obtain the unique irreversible ergodic Markov transition matrix and its invariant measure having maximum entropy among those consistent with the constraints provided by data. This approach solves the problem of the bad estimations mentioned above and enables us to compute the IEP of the inferred Markov process, which is our main goal.

### 3.1. Inference of the Maximum Entropy Markov Process

The problem of estimating the Markov chain of maximum entropy constrained by the data is of general interest in information theory. It consists in solving a constrained maximization problem, from which one builds a Markov chain. The first step is choosing (arbitrarily) a set of indicator functions (also called monomials) and determine from the data the empirical average of these functions. This fixes the constraints of the maximization problem. After that, one maximizes the information entropy rate, which is a concave functional in the space of Lagrange multipliers associated to the constraints, obtaining the unique Markov measure that better approximates the statistics among all probability measures that match exactly the constraints [23]. To to our knowledge, previous approaches ignore how to deal with the inference of irreversible Markov processes in the maximum entropy context [37,38].

### 3.2. Observables and Potentials

Let us consider the space of infinite binary sequences $\Sigma_{N}$. An *observable* is a function $f:\Sigma_{N}\to R$. We say that an observable *f* has *range R* if it depends only on *R* consecutive spike patterns, e.g., $f\left(\sigma \right)=f\left(\sigma^{0,R-1}\right)$. We consider here that observables do not depend explicitly on time (*time-translation invariant observables*), i.e., for any time-step *n*, $f\left(\sigma^{0,R-1}\right)=f\left(\sigma^{n,n+R-1}\right)$ whenever $\sigma^{0,R-1}=\sigma^{n,n+R-1}$. Examples of observables are products of the form:

(12) $$f\left(\sigma^{0,T}\right)=\prod_{u=1}^{r}\sigma_{k_{u}}^{n_{u}},$$

where $k_{u}=1,\cdots ,N$ (neuron index) and $n_{u}=0,\cdots ,T$ (time index). These observables are called *monomials* and take values in {0,1}. Typical choices of monomials are $\sigma_{k_{1}}^{n_{1}}$ which is 1 if neuron $k_{1}$ fires at time $n_{1}$ and 0 otherwise; $\sigma_{k_{1}}^{n_{1}}\;\sigma_{k_{2}}^{n_{2}}$ which is 1 if neuron $k_{1}$ fires at time $n_{1}$ and neuron $k_{2}$ fires at time $n_{2}$ and 0 otherwise. For *N* neurons and time range *R* there are $2^{NR}$ possible monomials. To alleviate notations, instead of labeling monomials by a list of pairs, as in (12), we label them by an integer index, *l* (the index is defined in the same way as the block index (1)), i.e., a monomial reads $m_{l}$.

A *potential* is an observable that can be written as a linear combination of monomials (the range of the potential is the maximum over the ranges of the $m_{l}$ monomials considered). A potential of range *R* is written as follows:

(13) $$H\left(\sigma^{\left(\ell \right)}\right)\;:=\;\sum_{l=1}^{2^{NR}}h_{l}m_{l}\left(\sigma^{\left(\ell \right)}\right)\;\sigma^{\left(\ell \right)}\in \Sigma_{N}^{R},$$

where the coefficients $h_{l}$ real numbers. Some coefficients in this series may be zero. We assume throughout this paper that $h_{\ell }<\infty$ (here, we do not consider hard core potentials with forbidden configurations). One example of potential is the one considering as monomials the firing rates $\sigma_{i}$ and the synchronous pairwise correlations $\sigma_{i}\;\sigma_{j}$.

$$H\left(\sigma^{\left(\ell \right)}\right)=\sum_{i=1}^{N}h_{i}\sigma_{i}+\frac{1}{2}\sum_{i,j=1}^{N}J_{ij}\sigma_{i}\;\sigma_{j}\;\sigma^{\left(\ell \right)})\in \Sigma_{N}^{1}$$

#### Additive Observables of Spike Trains

Let ϕ be the shift map $\phi :\Sigma_{N}\to \Sigma_{N}$, defined by $\phi {\left(\sigma \right)}_{\left(i\right)}=\sigma_{\left(i+1\right)}$. Let *f* be an arbitrary observable. We may consider the sequence $\left\{f\circ \phi^{i}\left(\sigma \right)\right\}$ as a random variable whose statistical properties depend on those of the process producing the samples of σ and the regularity of the observable *f*.

Given a spike train, one would like to empirically quantify properties of empirical averages and their fluctuation properties as a function of the sampling size. Consider a spike train σ, and let *n* be the sample length. The average of the observable *f* of range R≥1 in σ is given by,

$$A_{n}\left(f\right)=\frac{1}{n-R+1}\sum_{i=0}^{n-R}f\circ \phi^{i}\left(\bar{\sigma }\right),$$

in particular, for observables of range 1, one has

(14) $$A_{n}\left(f\right)=\frac{1}{n}\sum_{i=0}^{n-1}f\left(\sigma^{i}\right).$$

### 3.3. Variational Principle

Let $A_{n}\left(f_{k}\right)=C_{k}$ be the average value of *K* observables for k∈{1,⋯,K}. As the empirical average of monomials is not enough to uniquely determine the spike train statistics (there are infinitely many probability measures sharing the same averages of monomials), we use the maximum entropy method to obtain the Markov measure μ that maximizes the KSE among all measures ν that match the expected values of all observables, i.e., $\nu \left[f_{k}\right]=C_{k}$, for all k∈{1,⋯,K}. This is equivalent to solve the following variational problem under constraints:

(15) $$S\left[\;\mu \;\right]=\max\left\{S\left[\;\nu \;\right]:\nu \left[\;f_{k}\;\right]=C_{k}\;\forall \;k\in \left\{1,\cdots ,K\right\}\right\}.$$

Since the function $\nu \to S\left[\;\nu \;\right]$ is strictly concave, there is a unique maximizing Markov measure μ(π,P) given the set of values $C_{k}$. To solve this problem, we introduce the set of Lagrange multipliers $h_{k}\in R$ in the potential $H=\sum_{k=1}^{K}h_{k}f_{k}$, which is a linear combination of the chosen observables. Next, we study the following unconstrained problem, which is a particular case of the so-called *variational principle* of the thermodynamic formalism [36]:

(16) $$P\left[\;H\;\right]=\underset{\nu \in M_{inv}}{\sup}\left\{S\left[\;\nu \;\right]\;+\;\nu \left[\;H\;\right]\right\}=S\left[\;\mu \;\right]\;+\;\mu \left[\;H\;\right],$$

where $P\left[\;H\;\right]$ is called the *free energy or topological pressure*, $M_{inv}$ is the set of invariant measures with respect to the shift ϕ and $\nu \left[\;H\;\right]=\sum_{k=1}^{K}h_{k}\;\nu \left[\;f_{k}\;\right]$ is the average value of H with respect to ν.

In this paper, we only consider potentials H of finite range, for which there is a unique measure μ attaining the supremum [39] and is a *Gibbs measure in the sense of Bowen.*

*Gibbs measures in the sense of Bowen*: Suppose H is a finite range potential R≥2, a shift invariant probability measure μ is called a Gibbs measure (in the sense of Bowen) if there are constants M>1 and P[H]∈R s.t.

(17) $$M^{-1}\leq \frac{\mu [\sigma^{1,n}]}{\exp(\sum_{k=1}^{n-R+1}H\left(\sigma^{k,k+R-1}\right)-\left(n+R-1\right)P\left[H\right])}\leq M$$

It is easy to see that the classical form of Boltzmann–Gibbs distributions $\mu \left[\sigma \right]=e^{H(\sigma )}/Z$ is a particular case of (17), when M=1,H is a potential of range R=1 and P[H]=logZ.

#### Statistical Inference

The functional $P\left[\;H\;\right]$ has the following property:

(18) $$\frac{\partial P\left[\;H\;\right]}{\partial h_{k}}=\mu \left[f_{k}\right]=C_{k},\;\forall k\in \left\{1,\ldots ,K\right\}$$

where $\mu [f_{k}]$ is the average of $f_{k}$ with respect to μ, which is equal to the average value of $f_{k}$ with respect to the empirical measure from the data $C_{k}$, by constraint of the maximization problem. For finite range potentials, P(H) is a convex function of $h_{l}$’s. This ensures the uniqueness of the solution of (16). Efficient algorithms exist to estimate the Lagrange multipliers for the maximum entropy problem with non-synchronous constraints [18].

### 3.4. Ruelle–Perron–Frobenius Transfer Operator

Consider H to be an arbitrary potential, and *w* a continuous function on $\Sigma_{N}$. We introduce the *Ruelle–Perron–Frobenius* (R–P–F) transfer operator denoted by $L_{H}$, and it is given by,

$$L_{H}w\left(\sigma \right)=\sum_{\sigma^{\prime }\in \Sigma_{N},\phi \left(\sigma^{\prime }\right)=\sigma }e^{H(\sigma^{\prime })}w\left(\sigma^{\prime }\right).$$

In an analogous way, as it is done for Markov approximations of Gibbs measures [40,41], for a finite range potential H, we introduce the *transfer matrix $L_{H}$,*

(19) $$L_{H}\left(\ell ,\ell^{\prime }\right)=\left\{\begin{array}{cc} e^{H(\sigma^{0,L})}\; & \mathrm{if}\sigma^{0,L}∼\sigma^{\left(\ell \right)}\to \sigma^{\left(\ell^{\prime }\right)}\\ 0,\; & \mathrm{otherwise}. \end{array}\right.$$

From the assumption H>−∞, each allowed transition corresponds to a positive entry in the matrix $L_{H}$.

### 3.5. Maximum Entropy Markov Chain for Finite Range Potentials

The matrix (19) is primitive (the matrix *A* is primitive if there is an n∈N, s.t. $A^{n}$ has only positive components) by construction, thus it satisfies the Perron–Frobenius theorem [42]. Let ρ>0 be its spectral radius. Because of the irreducibility of the transfer matrix, ρ is an eigenvalue of multiplicity 1 strictly larger in modulus than the other eigenvalues. For every $\sigma^{\left(\ell \right)}\in \Sigma_{N}^{L}$, let us denote by $L_{\ell }:=L\left(\sigma^{\left(\ell \right)}\right)$ and $R_{\ell }:=R\left(\sigma^{\left(\ell \right)}\right),$ the left and right eigenvectors of $L_{H}$ corresponding to the eigenvalue ρ. Notice that $L_{\ell }>0$ and $R_{\ell }>0$ for all $\sigma^{\left(\ell \right)}\in \Sigma_{N}^{L}$. Using spectral properties of the transfer matrix, we get the maximum entropy Markov transition probability matrix [39]:

(20) $$P_{\ell ,\ell^{\prime }}:=\frac{L_{H}\left(\ell ,\ell^{\prime }\right)R_{\ell^{\prime }}}{R_{\ell }\;\rho },\;\forall \sigma^{\left(\ell \right)},\sigma^{\left(\ell^{\prime }\right)}\in \Sigma_{N}^{L}.$$

The unique stationary probability measure π associated to *P* is also obtained by the spectral properties of $L_{H}$:

(21) $$\pi_{\ell }:=\frac{L_{\ell }\;R_{\ell }}{\langle L,R\rangle },\;\forall \sigma^{\left(\ell \right)}\in \Sigma_{N}^{L}.$$

For a finite range potential H, the unique measure μ(π,P) associated to H, satisfies the variational principle and, furthermore, the topological pressure can be explicitly computed P[H]=lnρ.

### 3.6. IEP of the Inferred Markov Maximum Entropy Process

Consider a potential H of finite range and the state space $\Sigma_{N}^{L}$. As we have seen before, using the maximum entropy framework one can build from the transfer matrix $L_{H}$, the Markov transition matrix *P* and its invariant measure π. Furthermore, one can apply straightforwardly (20) and (21) to obtain a formula for the IEP based only on the spectral properties of $L_{H}$. After simplifying we get:

(22) $$IEP\left(L_{H}\right)=\sum_{\sigma^{\left(\ell \right)},\sigma^{\left(\ell^{\prime }\right)}\in \Sigma_{N}^{L}}\frac{L_{\ell }}{\langle L,R\rangle }\frac{L_{H}\left(\ell ,\ell^{\prime }\right)R_{\ell^{\prime }}}{\rho }\log\left[\frac{L_{\ell }\;R_{\ell^{\prime }}\;L_{H}\left(\ell ,\ell^{\prime }\right)}{L_{\ell^{\prime }}\;R_{\ell }\;L_{H}\left(\ell^{\prime },\ell \right)}\right]$$

This is a quantity of major interest in spike train statistics, as it measures the degree of time irreversibility of the inferred maximum entropy Markov chain. Although it is a straightforward result, it is quite general and of practical use, as we will see in the examples below.

We can apply (20) and (21) to Equation (7), we obtain the detailed balance condition in terms of the transfer matrix and its spectral properties:

$$\frac{L_{\ell }\;R_{\ell }}{\langle L,R\rangle }\frac{L_{H}\left(\ell ,\ell^{\prime }\right)\;R_{\ell^{\prime }}}{R_{\ell }\;s}=\frac{L_{\ell^{\prime }}\;R_{\ell^{\prime }}}{\langle L,R\rangle }\frac{L_{H}\left(\ell^{\prime },\ell \right)\;R_{\ell }}{R_{\ell^{\prime }}\;s}$$

Simplifying, we obtain:

(23) $$\frac{L_{H}\left(\ell ,\ell^{\prime }\right)}{L_{H}\left(\ell^{\prime },\ell \right)}=\frac{R_{\ell }\;L_{\ell^{\prime }}}{R_{\ell^{\prime }}\;L_{\ell }}$$

### 3.7. Large Deviations for Observables of Maximum Entropy Markov Chains

The goal of large deviations is to compute the asymptotic probability distribution $P(A_{n}\left(f\right)=s)$ for a given finite range observable *f* and for s≠E(f). More precisely, we say that $P(A_{n}\left(f\right))$ satisfies a large deviation principle with rate $I_{f}\left(s\right)$ if the following limit exists,

$$\lim_{n\to \infty }-\frac{1}{n}\ln P\left(A_{n}\left(f\right)=s\right)=I_{f}\left(s\right).$$

where the dominant behavior of $P(A_{n}\left(f\right))$ is decaying exponentially fast with the sample size *n*, as

(24) $$P\left(A_{n}\left(f\right)=s\right)\approx e^{-nI_{f}\left(s\right)}.$$

We define the *scaled cummulant generating function* (SCGF) associated to the random variable (observable) *f* denoted by $\lambda_{f}\left(k\right)$ as follows,

(25) $$\lambda_{f}\left(k\right):=\lim_{n\to \infty }\frac{1}{n}\ln E\left[e^{nkA_{n}\left(f\right)}\right],\;k\in R.$$

The *n*-th cumulant of the random variable *f* can be obtained by differentiating $\lambda_{f}\left(k\right)$ with respect to *k*, *n* times and evaluating the result at k=0. The next theorem by Gärtner–Ellis theorem relates the SCGF and the large deviations rate function. The Gärtner–Ellis theorem relies on the differentiability of $\lambda_{f}\left(k\right)$, which is guaranteed for finite state Markov chains [43]. This theorem has several formulations, which usually require some technical definitions beforehand. Here, we stated it in a simplified form, which is what we need for our purposes.

*Gärtner–Ellis theorem:* If $\lambda_{f}\left(k\right)$ is differentiable, then there exist a large deviation principle for the average process $A_{n}\left(f\right)$ whose rate function $I_{f}\left(s\right)$ is the Legendre transform of $\lambda_{f}\left(k\right)$:

(26) $$I_{f}\left(s\right)=\max_{k\in R}\left\{ks-\lambda_{f}\left(k\right)\right\}$$

The Gärtner–Ellis Theorem is very useful in our context, because it bypasses the direct calculation of $P(A_{n}\left(f\right))$ in (24), i.e., having $\lambda_{f}\left(k\right)$ a simple calculation leads to the rate function of *f*. As we will see in the next section $\lambda_{f}\left(k\right)$ naturally appears in the context of Maximum entropy Markov chains.

### 3.8. Large Deviations for the IEP

Consider an irreducible Markov chain with transition matrix $P_{\ell ,\ell^{\prime }}$. We define the *tilted transition matrix by f* denoted by ${\tilde{P}}^{\left(f\right)}\left(k\right)$, whose elements for a one time step observable are:

(27) $${\tilde{P}}_{\ell ,\ell^{\prime }}^{\left(f\right)}\left(k\right)=P_{\ell ,\ell^{\prime }}e^{kf(\ell^{\prime })}$$

or for a two time step observable:

(28) $${\tilde{P}}_{\ell ,\ell^{\prime }}^{\left(f\right)}\left(k\right)=P_{\ell ,\ell^{\prime }}e^{kf(\ell ,\ell^{\prime })}$$

For a Markov transition matrix *P* inferred from the maximum entropy, the tilted transition matrix can be built directly from the transfer matrix and its spectral properties.

(29) $${\tilde{P}}_{\ell ,\ell^{\prime }}^{\left(f\right)}\left(k\right)=\frac{L_{H}\left(\ell ,\ell^{\prime }\right)R_{\ell^{\prime }}}{R_{\ell }\;\rho }e^{kf(\ell ,\ell^{\prime })}$$

The Markov chain structure underlying $A_{n}\left(f\right)$ can be used here to obtain more explicit expressions for $\lambda_{f}\left(k\right)$. In the case of the additive observables, if a Markov chain is homogeneous and ergodic can compute explicitly the SCGF as the logarithm of the maximum eigenvalue of ${\tilde{P}}^{\left(f\right)}$:

(30) $$\lambda_{f}\left(k\right)=\ln\left(\rho \left({\tilde{P}}^{\left(f\right)}\right)\right)$$

This result is valid if the state-space of the Markov chain is finite, where it can be furthermore proven that $\lambda_{f}\left(k\right)$ is differentiable and $\lambda_{f}^{\prime }\left(0\right)=E\left(f\right)$.

**Remark** **1.** The observable f does not need to belong in the set ${\left\{f_{k}\right\}}_{k=1}^{K}$ of chosen observables to fit the Markov maximum entropy process. We denote $\rho ({\tilde{P}}^{\left(f\right)})$ the dominant eigenvalue (i.e., with largest magnitude) of the matrix ${\tilde{P}}^{\left(f\right)}$, which is unique by the Perron–Frobenius theorem.

We are interested in the fluctuations of the IEP. For that purpose, we define the following observable:

$$W_{n}\left({\left\{\sigma^{i}\right\}}_{i=1}^{n}\right)=\ln\left[\frac{P({\left\{\sigma^{i}\right\}}_{i=1}^{n})}{P({\left\{\sigma^{i}\right\}}^{\left(R\right)})}\right]$$

where ${\left\{\sigma^{i}\right\}}^{\left(R\right)}=\sigma^{n},\sigma^{n-1},\cdots ,\sigma^{1}$ is the temporal inversion of the trajectory ${\left\{\sigma^{i}\right\}}_{i=1}^{n}$. It can be shown that for P-almost every trajectory of a stationary ergodic Markov chain (π,P):

$$\lim_{n\to \infty }\frac{W_{n}\left({\left\{\sigma^{i}\right\}}_{i=1}^{n}\right)}{n}=IEP\left(\pi ,P\right)$$

It can be shown [28] that the SCGF $\lambda_{W}\left(k\right)$ associated to the observable $W_{n}$ can be found as the logarithm of the maximum eigenvalue ρ(k) of the matrix:

$${\tilde{P}}_{\ell ,\ell^{\prime }}^{\left(W\right)}\left(k\right)=P_{\ell ,\ell^{\prime }}e^{kF_{\ell ,\ell^{\prime }}}$$

where,

$$F_{\ell ,\ell^{\prime }}=\ln\left[\frac{\pi_{\ell }P_{\ell ,\ell^{\prime }}}{\pi_{\ell^{\prime }}P_{\ell^{\prime },\ell }}\right]$$

which is a matrix of positive elements.

Using the Gärtner–Ellis theorem, we obtain the rate function $I_{W}\left(s\right)$ for the IEP observable:

$$I_{W}\left(s\right)=\max_{k}\left\{ks-\lambda_{W}\left(k\right)\right\}$$

The rate function of the IEP observable has the following property:

$$\lambda_{W}\left(k\right)=\lambda_{W}\left(-k-1\right)$$

Since $\lambda_{W}^{\prime }\left(0\right)=IEP\left(\pi ,P\right)$ the symmetry implies

$$I_{W}\left(s\right)=I_{W}\left(-s\right)-s$$

#### Gallavotti–Cohen Fluctuation Theorem

The Gallavotti–Cohen fluctuation theorem refers to a symmetry in the fluctuations of the IEP. It is a statement about the large deviations of $\frac{W_{n}}{n}$, which is the time-averaged entropy production rate of the sample trajectory ${\left\{\sigma^{i}\right\}}_{i=1}^{n}$ of the Markov chain μ(π,P).

$$\frac{P\left[\frac{W_{n}}{n}\approx s\right]}{P\left[\frac{W_{n}}{n}\approx -s\right]}≍e^{ns}$$

This means that the positive fluctuations of $\frac{W_{n}}{n}$ are exponentially more probable than negative fluctuations of equal magnitude. This is a universal ratio, i.e., no free parameters are involved and is experimentally observable.

## 4. Examples

In this section, we provide several examples of applications of our results in the context of spike train statistics. We first provide an example of a discrete time Integrate-and-fire (IF) neuronal network model. This example does not use the MEP as the transition matrix can be explicitly obtained from the dynamics of the model. We then come back to the MEP approach to characterize the spike train statistics and compute the IEP for each example. We finally provide a summary of the results and discuss our findings.

### 4.1. Example: Discrete Time Spiking Neuronal Network Model

The IF model is one of the most ubiquitous models to simulate and analyze the dynamics of spiking neuronal circuits. This model is the simplest dynamical model that captures the basic properties of neurons, including the temporal integration of noisy sub-threshold inputs and all-or-nothing spiking. At the level of networks postulates a set of equations describing the behavior of the interconnected neurons motivated by the microscopic picture of how the biological neuronal network is supposed to work.

There exist several different versions of this model. Here, we present the discrete time IF model. The model definition follows the presentation given in [44]. Neurons are considered as points, without spatial extension nor biophysical structure (axon, soma, and dendrites). The dynamical system is only ruled by discrete time dynamical variables.

Denote by V(t) the membrane potential vector with entries $V_{i}\left(t\right)$, whose dynamics is defined as follows. Fix a real variable θ>0 called *firing threshold*. For a fixed discrete time *t*, we have two possibilities:

- $V_{i}\left(t\right)<\theta$, for all k=1,…,N. This corresponds to sub-threshold dynamics.
- There exists a *k* such that, $V_{k}\left(t\right)\geq \theta$. Corresponding to firing dynamics.

The under-threshold dynamics is given by the following equation:

(31) $$V\left(t+1\right)=F\left(V\left(t\right)\right)+\sigma_{B}B\left(t\right)$$

where

(32) $$F_{i}\left(V\left(t\right)\right)=\gamma V_{i}\left(t\right)\left(1-Z[V_{i}\left(t\right)]\right)+\alpha \sum_{j=1}^{N}W_{ij}Z\left[V_{j}\left(t\right)\right]+\beta I_{i}.$$

The function $Z\left[x\right]:=1_{x\geq \theta }$ is called the *firing state* of neuron *x*, where 1 is the indicator function. When $Z[V_{i}\left(t\right)]=1$ one says that neuron *i* *spike*, otherwise is *silent*. We extend the definition of *Z* to vectors: Z[V(t)] is the vector with components $Z[V_{i}\left(t\right)],i=1,\ldots ,N$. The *leak rate* is denoted by γ∈[0,1], and $W_{ij}$ is called the *synaptic weight* from the neuron *j* to the neuron *i*. The synaptic weight is said to be *excitatory* if $W_{ij}>0$ or *inhibitory* if $W_{ij}<0$. The components of the vector B(t) are independent normalized Gaussian random variables and $\sigma_{B}$ is the noise amplitude parameter. The parameters α and β are introduced in order to control the intensity of the synaptic weights and the stimulus, respectively.

From this model, one can obtain a set of conditional probabilities of spike patterns given the network’s spike history, allowing a mechanistic and causal interpretation of the origin of correlations (see [44] for details). Here, we consider only one time-step dependence on the past, although in the general approach it is possible to consider infinite memory. The conditional probabilities (transition matrix elements) are given as follows:

(33) $$P\left[\sigma |\sigma^{\prime }\right]=\prod_{i=1}^{N}\left[\sigma_{i}\;\varphi \left(\frac{\theta -C_{i}\left(\alpha ,\beta ,\sigma^{\prime }\right)}{\sigma_{B}}\right)+\left(1-\sigma_{i}\right)\left(1-\varphi \left(\frac{\theta -C_{i}\left(\alpha ,\beta ,\sigma^{\prime }\right)}{\sigma_{B}}\right)\right)\right],$$

where,

(34) $$C_{i}\left(\alpha ,\beta ,\sigma^{\prime }\right)=\gamma \;\alpha \sum_{j=1}^{N}W_{ij}\sigma_{j}^{\prime }+\beta I_{i}$$

and

(35) $$\varphi \left(x\right)=\int_{x}^{\infty }e^{\frac{-u^{2}}{2}}du.$$

**Figure 1:**
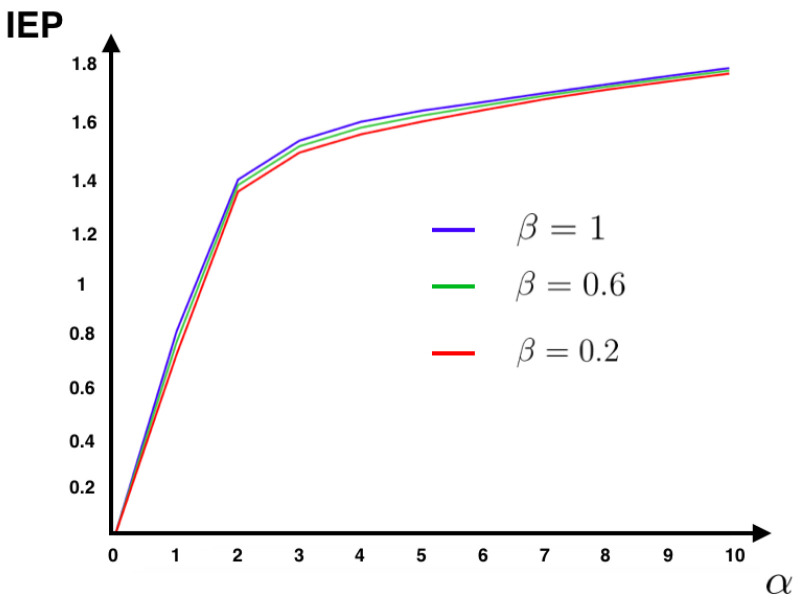
Plot of the average value of IEP for 500 realizations of the synaptic weight matrix for fixed α and β in each case. We fix the following values of the parameters: N=6, γ=0.2, $\sigma_{b}=1$, θ=1, $I_{i}=1$ ∀i∈{1,…,6}. The components of the synaptic weight matrix $W_{ij}$ were drawn at random from a normalized Gaussian distribution. We plot the average value of IEP for 500 realizations of the synaptic weight matrix for fixed α and β in each case.

The function *C* takes into account the past and the external stimuli (see [44] for details). These transition probabilities define an ergodic Markov chain specified by the biophysical dynamics of the spiking network. From the transition probabilities (33) and the unique steady state, we compute the IEP of this model using (11) for different values of the parameters α and β (see Figure 1).

Figure 1 shows that for this model the IEP depends mostly on the intensity of the synaptic weights, while the stimulus intensity is playing a minor role. This suggests that IEP (in the stationary case) is essentially a property of the spiking neuronal network structure. The IEP of this neuronal network model is zero only under very restricted and unrealistic biophysical circumstances: when all synaptic weights are identical in amplitude and with the same sign or when they are all zero, i.e., when neurons do not communicate among them. In the first case, spikes play a symmetrical role with respect to time, which cancels out when computing the IEP. In the second case, the associated stochastic process is time-independent (thus time reversible). Therefore, generically this biophysically plausible model of spiking neuronal networks, has positive IEP. This means that the spike dynamics of this model leads to an irreversible Markov process.

### 4.2. MEMC Example: One Observable

In the previous example, we assume known the transition probabilities i.e., the structure of synaptic connectivity, stimulus and all other parameters defining the spiking neuronal network. Unfortunately, this is not always the case. Alternative approaches based on the MEP are considered when only spiking data are available. Consider a range-2 potential with N=2 neurons:

$$H\left(\sigma^{0,1}\right)=h_{1}\sigma_{1}^{1}\sigma_{2}^{0}.$$

The transfer matrix (19) associated to H is in this case a 4×4 matrix:

$$L_{H}=\left(\;\begin{array}{cccc} 1 & 1 & 1 & 1\\ 1 & 1 & 1 & 1\\ 1 & e^{h_{1}} & 1 & e^{h_{1}}\\ 1 & e^{h_{1}} & 1 & e^{h_{1}} \end{array}\;\right).$$

As this matrix is primitive by construction, it satisfies the hypothesis of the Perron–Frobenius theorem. The unique maximum eigenvalue is $\rho =e^{h_{1}}+3$. The left and right eigenvectors associated to this largest eigenvalue are respectively:

$$L\left(\;\begin{array}{c} 0\\ 0 \end{array}\;\right)=\frac{2}{1+e^{h_{1}}};\;L\left(\;\begin{array}{c} 0\\ 1 \end{array}\;\right)=1;\;L\left(\;\begin{array}{c} 1\\ 0 \end{array}\;\right)=\frac{2}{1+e^{h_{1}}};\;L\left(\;\begin{array}{c} 1\\ 1 \end{array}\;\right)=1,$$

$$R\left(\;\begin{array}{c} 0\\ 0 \end{array}\;\right)=\frac{2}{1+e^{h_{1}}};\;R\left(\;\begin{array}{c} 0\\ 1 \end{array}\;\right)=\frac{2}{1+e^{h_{1}}};\;R\left(\;\begin{array}{c} 1\\ 0 \end{array}\;\right)=1;\;R\left(\;\begin{array}{c} 1\\ 1 \end{array}\;\right)=1.$$

From the spectral properties of $L_{H}$, we obtain the Markov transition matrix (20), which reads:

$$P_{\sigma^{0},\sigma^{1}}=\frac{1}{\rho }\;\left(\;\begin{array}{cccc} 1 & 1 & \frac{1+e^{h_{1}}}{2} & \frac{1+e^{h_{1}}}{2}\\ 1 & 1 & \frac{1+e^{h_{1}}}{2} & \frac{1+e^{h_{1}}}{2}\\ \frac{2}{1+e^{h_{1}}} & \frac{2e^{h_{1}}}{1+e^{h_{1}}} & 1 & e^{h_{1}}\\ \frac{2}{1+e^{h_{1}}} & \frac{2e^{h_{1}}}{1+e^{h_{1}}} & 1 & e^{h_{1}} \end{array}\;\right),$$

The unique invariant measure of this irreducible Markov transition matrix is given by Equation (21), and its entries are given by:

$$\pi \left(\begin{array}{c} 0\\ 0 \end{array}\right)=\frac{4}{\rho^{2}},\;\pi \left(\begin{array}{c} 0\\ 1 \end{array}\right)=\frac{2(\rho -2)}{\rho^{2}},\;\pi \left(\begin{array}{c} 1\\ 0 \end{array}\right)=\frac{2(\rho -2)}{\rho^{2}},\;\pi \left(\begin{array}{c} 1\\ 1 \end{array}\right)=\frac{{\left(\rho -2\right)}^{2}}{\rho^{2}}.$$

It is easy to check that π is invariant w.r.t. the transition matrix *P*, that is πP=π.

From this example, we can verify that *generically the detailed balance condition is not satisfied*; for example:

$$P\left(\begin{array}{c} 0\\ 1 \end{array}\right|\begin{array}{c} 1\\ 0 \end{array})\pi \left(\begin{array}{c} 1\\ 0 \end{array}\right)\neq P\left(\begin{array}{c} 1\\ 0 \end{array}\right|\begin{array}{c} 0\\ 1 \end{array})\pi \left(\begin{array}{c} 0\\ 1 \end{array}\right).$$

**Figure 2:**
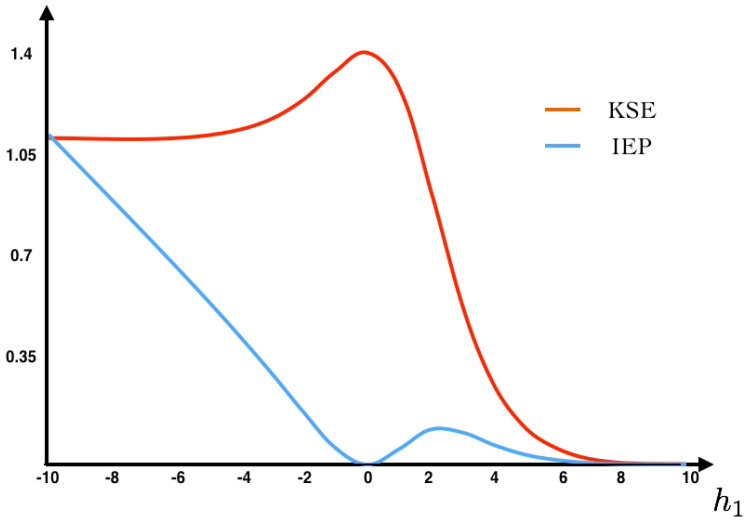
IEP and KSE as a function of $h_{1}$. In this example, the detailed balance condition is only satisfied in the trivial case $h_{1}=0$, corresponding to the uniform distribution. In all other cases, we obtain a MEMC with positive IEP, that is a NESS.

As we can see in Figure 2, the maximum entropy measure for the unconstrained problem is attained at the uniform distribution ($h_{1}=0$, eigenvalue ρ=4 assigning probability $\frac{1}{4}$ to each spike pattern).

Let us now consider a constrained version of this problem. Suppose we have a dataset of length *T* and we measure the average value of the observable considered in this example $f=\sigma_{1}^{1}\sigma_{2}^{0}$,

$$A_{T}\left(f\right)=c_{1}$$

Given this restriction and using the Equation (18), we obtain the following equation:

$$\frac{\partial \log(e^{h_{1}}+3)}{\partial h_{1}}=c_{1}$$

Solving we find $h_{1}$. Among all the Markov chains that match exactly the restriction, the one that maximizes the information entropy is the one obtained by fixing $h_{1}$ at the found value. Is easy to check that the variational principle (16) is satisfied.

**Figure 3:**
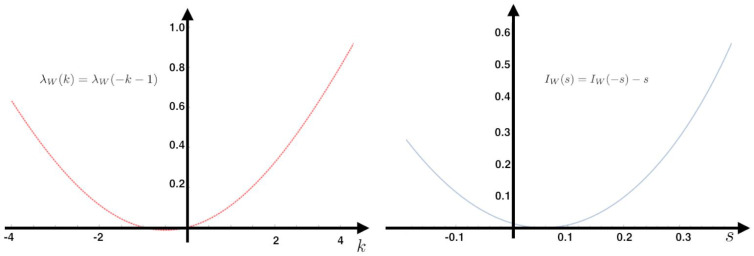
Gallavotti–Cohen fluctuation theorem for the MEMC example with one observable at the parameter value $h_{1}=-1$. Left: We show the SCGF associated to $W,\lambda_{W}\left(k\right)$, the derivative at zero is the IEP of the MEMC, which in this case is 0.0557. This value coincides with the minimum of the rate function $I_{W}\left(s\right)$ at the right side of the figure.

From the transition probability matrix *P* and the invariant measure π, we compute the KSE (Equation (9)) and the IEP (Equation (22)) as a function of the parameter $h_{1}$ (see Figure 2). Additionally, we fix the value $h_{1}=-1$ at which we compute the IEP. We also compute the fluctuations around the mean and the large deviations. The Gallavotti–Cohen theorem applied to this example is illustrated in Figure 3.

### 4.3. MEMC Example: Two Observables

Consider now a similar neural system of two interacting neurons, but now take into account two observables representing how one neuron influences the other in the next time step.

$$f_{1}\left(\sigma^{0,1}\right)=\sigma_{1}^{1}\sigma_{2}^{0}\;\mathrm{and}\;f_{2}\left(\sigma^{0,1}\right)=\sigma_{2}^{1}\sigma_{1}^{0}$$

From these two features, one can build the corresponding energy function

(36) $$H\left(\sigma^{0,1}\right)=h_{1}f_{1}\left(\sigma^{0,1}\right)+h_{2}f_{2}\left(\sigma^{0,1}\right),$$

where $h_{1},h_{2}$ are the parameters.

**Figure 4:**
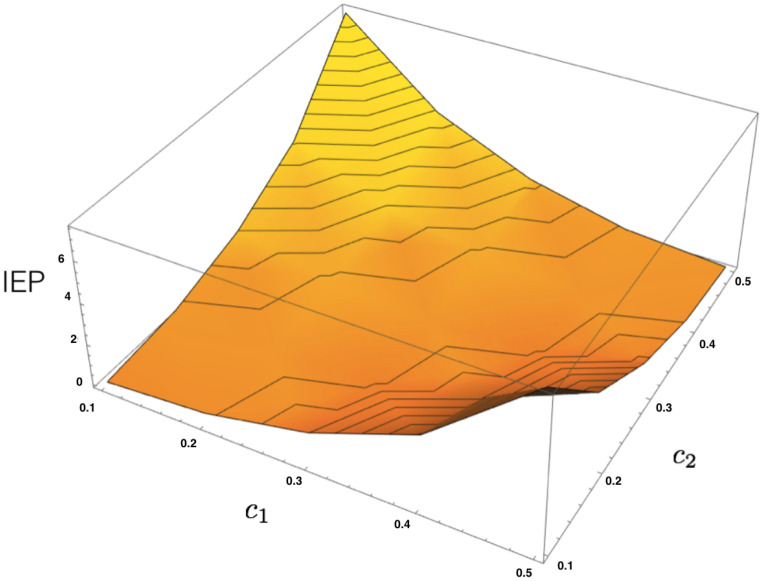
IEP for the MEMC build from each pair of constraints for the example Section 4.3. We use the restrictions on the average values denoted by $c_{1}$ and $c_{2}$ to build the corresponding MEMC in each case. We compute the IEP for each pair. In this figure, we illustrate that the IEP is zero only when both restrictions are equal and that the IEP increases with the difference in the restrictions.

Given a dataset, let us denote the corresponding empirical averages of both features as $A_{T}\left(f_{1}\right)=c_{1}$ and $A_{T}\left(f_{2}\right)=c_{2}$. From the energy function (36), we build the transfer matrix and apply the same procedure presented in the previous example to obtain the unique maximum entropy Markov transition matrix and the invariant measure to compute the IEP as a function of $c_{1}$ and $c_{2}$, as illustrated in Figure 4.

### 4.4. Example: Memoryless Potentials

Consider a finite and fix number of neurons *N* and a potential of range 1. This case includes the Ising model [10], Triplets [15], *K*-pairwise [25] and all other memoryless potentials that has been used in the context of maximum entropy models of spike train statistics. It represent a limit case in the definition of the transfer matrix. In this case, the potential does not “see” the past, i.e., $L_{H}\left(\sigma ,\sigma^{\prime }\right)=e^{H(\sigma^{\prime })}$. The matrix $L_{H}$ has a unique maximum eigenvalue:

$$\rho =Z=\sum_{\sigma^{\prime }\in \Sigma_{N}^{1}}e^{H(\sigma^{\prime })}$$

and the rest of eigenvalues are equal to 0. The left and right eigenvectors corresponding to ρ are:

$$L\left(\sigma^{\prime }\right)=\frac{1}{Z},\;R\left(\sigma^{\prime }\right)=e^{H(\sigma^{\prime })};\;\forall \sigma^{\prime }\in \Sigma_{N}^{1}.$$

Note that 〈L,R〉=1. We have therefore:

(37) $$P\left(\sigma^{\prime }|\sigma \right)=P\left(\sigma^{\prime }\right)=\pi \left(\sigma^{\prime }\right)=\frac{e^{H(\sigma^{\prime })}}{Z};\;\forall \sigma ,\sigma^{\prime }\in \Sigma_{N}^{1},$$

In this case, the invariant measure π has the classical Boltzmann–Gibbs form. The associated Markov chain has no memory: successive events are independent.

Taking the formula of IEP (22) we obtain:

$$IEP\left(L_{H}\right)=\sum_{\sigma ,\sigma^{\prime }\in \Sigma_{N}^{1}}\frac{L(\sigma )}{\langle L,R\rangle }\frac{e^{H(\sigma^{\prime })}R\left(\sigma^{\prime }\right)}{\log(Z)}\left(H\left(\sigma^{\prime }\right)-H\left(\sigma \right)\right)=0.$$

In the case where only range 1 observables are chosen, the average value of these observables in a given data set is the same as the one taken from another data set where the time indexes have been randomly shuffled or even time reversed. As this is the only information about the process that the maximum entropy principle consider, it is not surprising that the stochastic process associated with the maximum entropy measure is time reversible. Consider a data set consisting in binary patterns $D^{O}$. Let g:{0,⋯,T}→{0,⋯,T} be a function that randomly shuffles the time indexes, we call $D^{RS}$ the data set obtained after this transformation. Finally consider $D^{I}$, the data set with inverted time indexes,

$$\begin{array}{cc} D^{O} & =\{\sigma^{0},\sigma^{1},\sigma^{2},\cdots ,\sigma^{T-1},\sigma^{T}\}\\ D^{RS} & =\{\sigma^{g(0)},\sigma^{g(1)},\sigma^{g(2)},\cdots ,\sigma^{g(T-1)},\sigma^{g(T)}\}\\ D^{I} & =\{\sigma^{T},\sigma^{T-1},\sigma^{T-2},\cdots ,\sigma^{1},\sigma^{0}\}. \end{array}$$

**Figure 5:**
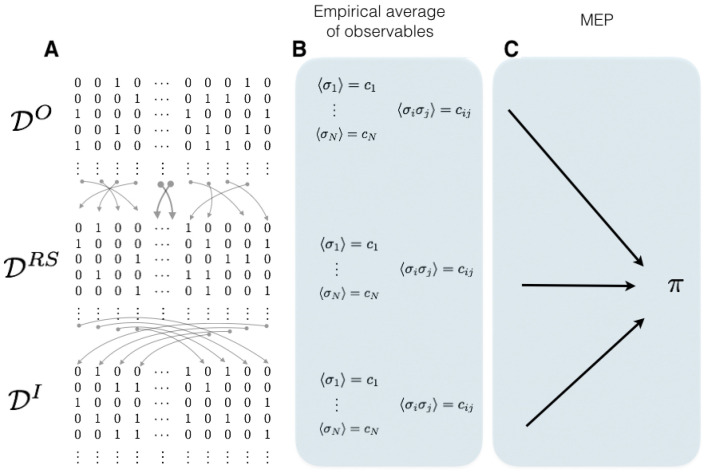
Memoryless potentials do not distinguish shuffled nor time-inverted data sets. Illustrative scheme showing three different data sets sharing the same maximum entropy distribution π. (**A**) We illustrate three data sets: on the top is the original; in the middle is the one obtained by randomly shuffle the time-indexes of the spike patterns; and on the bottom is the data set obtained by inverting the time indexes. (**B**) For each of these datasets, we compute the firing rate of each neuron denoted by $\langle \sigma_{i}\rangle$ and the pairwise correlations $\langle \sigma_{i}\sigma_{j}\rangle$ obtaining for each data set the same average values. (**C**) The spike train statistics of these three data sets are characterized by the same time-independent maximum entropy distribution π.

Observe that, in these three cases (that may correspond to very different biological experiments), the average value of every observable of range one is exactly the same, therefore these data sets are characterized by the same maximum entropy distribution as illustrated in Figure 5.

### 4.5. Example: 1-Time Step Markov with Random Coefficients

Here, we consider the 1-time step extension of the Ising model, that reads:

(38) $$H\left(\sigma^{0,1}\right)=\sum_{i=1}^{N}h_{i}\sigma_{i}+\frac{1}{2}\sum_{i,j=1}^{N}J_{ij}\sigma_{i}\;\sigma_{j}+\sum_{i,j=1}^{N}\gamma_{ij}\sigma_{i}\;\sigma_{j}^{1}.$$

**Figure 6:**
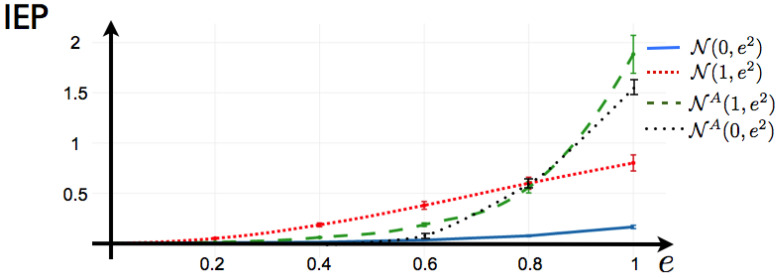
IEP for the 1-time step Markov potential. The parameters $h_{i}$ and $J_{ij}$ are draw at random one time and remain fixed. We draw at random the components of 100 matrices $\gamma_{ij}$ from a Gaussian distribution with different values of mean and standard deviation *e*. We plot the average value of IEP for each case, with the respective error bars.

This is the potential considered to fit a maximum entropy distribution to spiking data from a mammalian parietal cortex in-vivo in [20]. It is important to notice that in [20], the authors compute the solution of the maximum entropy problem imposing detailed balance condition, so in their case, there is zero IEP by construction. Here, we do not consider a particular data set, instead we investigate the capability of this potential to generate IEP by considering the following scenarios: We consider a network of N=10 neurons, where we draw at random the coefficients $h_{i}$ and $J_{ij}$ in a range plausible to be the maximum entropy coefficients (or Lagrange multipliers) of an experiment of retinal ganglion cells exposed to natural stimuli (values of from $h_{i}$ and $J_{ij}$ as in [26]). We generate the matrix $\gamma_{ij}$ by drawing each component at random from Gaussian distributions with different means and standard deviations. We summarize our results in Figure 6. We observe the following: Independent of $h_{i}$ and $J_{ij}$ and the parameters of mean and variance from which the matrix of coefficients $\gamma_{ij}$ is generated, if $\gamma_{ij}$ is symmetric, the Markov process generated by the potential (38) is reversible in time so the IEP is zero. This includes the limit case when $\gamma_{ij}=0,\forall i,j\in \left\{1,\cdots ,N\right\}$, where we recover the Ising model. Next, we fix the values of $h_{i}$ and $J_{ij}$ (random values), and we generate 100 matrices $\gamma_{ij}$ by drawing their components from Gaussian distributions $N(0,e^{2})$, another 100 from $N(1,e^{2})$. We also generate 100 anti-symmetric matrices $\gamma_{ij}$ from $N(1,e^{2})$, that we denote in Figure 6 $N^{A}\left(1,e^{2}\right)$. For each realization of $\gamma_{ij}$, we generate the transfer matrix and proceed as explained in Section 3 to obtain the IEP in each case.

Figure 6 shows that for fitted data with a maximum entropy 1-time step Markov model, the IEP is zero only when all the measured 1-step correlations between neurons are symmetric, which is very unlikely for an experimental spike train. The degree of symmetry in the matrix of γ’s play an important role in the IEP.

### 4.6. Example: Kinetic Ising Model with Random Asymmetric Interactions

This model of spike generation is an example of a non-equilibrium system, which has been used in [45] to approach the question of recovering the interactions of an asymmetrically-coupled Kinetic Ising model, with a time-independent external field to ensure stationarity. This is a discrete-time, synchronously updated Markov model in $\Sigma_{N}^{1}$ with transition matrix is given by:

(39) $$P\left[\sigma^{\prime }|\sigma \right]=\prod_{i=1}^{N}\frac{\exp[\left(2\sigma_{i}^{\prime }-1\right)\theta_{i}\left(\sigma \right)]}{2\cosh[\theta_{i}\left(\sigma \right)]},\;\forall \sigma ,\sigma^{\prime }\in \Sigma_{N}^{1}$$

(40) $$\theta_{i}\left(\sigma \right)=\beta \;h_{i}+\alpha \;\sum_{j=1}^{N}J_{ij}\left(2\sigma_{i}-1\right)\;\forall \sigma \in \Sigma_{N}^{1}.$$

The fields $h_{i}$ and the couplings $J_{ij}$ are independent Gaussian variables and α,β∈R. These set of stationary transition probabilities characterize an ergodic Markov chain with a unique invariant measure. With these two quantities at hand, the scene is set to compute information entropy production under different scenarios.

**Figure 7:**
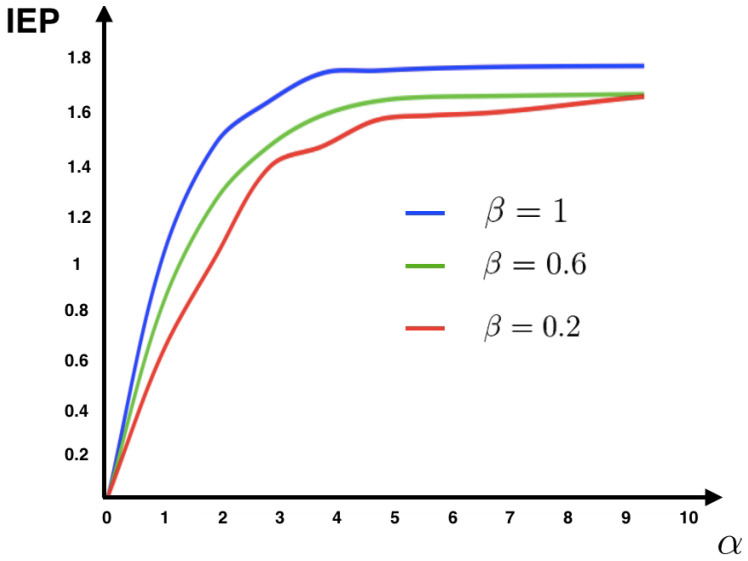
IEP for the Kinetic Ising model with random asymmetric interactions. We consider N=6. The components of field vector were drawn at random from a Gaussian N(−3,1) and the coupling matrix $J_{ij}$ were drawn at random from a Gaussian N(0,1). We plot the average value of IEP for 500 realizations of the synaptic coupling matrix for fixed α and β in each case.

In Figure 7, we recover the same structure found in Figure 1 for the IF model. This fact suggests that in this model the synaptic couplings are playing a major role in IEP, while the intensity of the stimulus is less relevant.

### 4.7. Summary

We have shown several examples of applications of our results in the context of spike train statistics. We first provide an example of a discrete time Integrate-and-fire (IF) neuronal network model to illustrate that, in multiple scenarios of synaptic connectivity, even with constant stimulus, we find positive IEP (see Figure 2). This example does not use the MEP as the transition matrix can be explicitly obtained from the dynamics of the model. We use this example to illustrate that time irreversible statistical models arise naturally from biologically realistic spiking neuronal network models and to emphasize that IEP can be obtained from this approach as a benchmark for the MEP approach. We then consider the MEP approach to characterize the spike train statistics. In the second example, we detail the transfer matrix technique to compute the maximum entropy Markov transition matrix and the invariant measure, from these two quantities the IEP is easily computed using (11). We illustrate the Gallavotti–Cohen fluctuation theorem of the IEP for this example (see Figure 3). The third example is used to illustrate how unlikely is to find a reversible MEMC from data as a strong condition $c_{1}=c_{2}$ need to be satisfied as shown in Figure 4. The fourth example is presented to show that our framework is general enough to consider the memoryless scenario as a limit case producing zero IEP. We illustrate in Figure 5 that memoryless maximum entropy models are not capable to distinguish among very different datasets. In the fifth example, we consider a MEMC, which has been considered in the literature of this field imposing detailed balance. We simulate different scenarios for the inter-neuron temporal parameters to illustrate the capability of this approach to capture the time-irreversible character of the underlying spiking network (see Figure 6). In the last example, we consider a popular model in the literature of this field to compute the IEP. Surprisingly, we recover the same structure of IEP (see Figure 7) as in the IF example (see Figure 1).

## 5. Discussion

The aim of population spike train statistical analysis is to deduce the principles of operation of neuronal populations. When trying to characterize the spike train statistics of networks of spiking neurons using the MEP, one hopes that the fitted parameters shed light on the understanding of some aspects of the population spiking phenomena in all its complexity. Therefore, to include and quantify time order in neuronal populations becomes a compulsory step towards a deeper understanding of the correlations observed in experimental data and consequently to better understand some aspects of the population neural code. The main message of this work is that limiting the complexity of the maximum entropy model using arguments of parsimony may not be appropriate to model a complex underlying stochastic process.

One of the consequences of including non-synchronous constraints in the MEP framework is that opens the possibility to broke the time-reversal symmetry imposed by time-independent models, and consequently to capture the irreversible character of the underlying biological process, allowing in this way to fit statistical models biologically more realistic. We have emphasized that the IEP is zero for time-independent processes (time-reversible) derived from commonly used statistical models in this field, for example, Ising, *K*-pairwise, triplets, among others [10,26]. However, *only time-dependent maximum entropy models induce time irreversible processes*, feature highly expected from biological systems.

While many spiking neuronal network models as the IF or the Generalized Linear Model (GLM) consider the influence of spike events occurred in the past, the most popular maximum entropy models in this field ignore them, causing a clear phenomenological disagreement between these two approaches, which can be corrected including non-synchronous constraints [22]. Leaving aside the fact that biophysical quantities used to fit realistic spiking neuronal network models may be difficult to obtain experimentally, the IEP obtained from both approaches to characterize the same neuronal tissue should be the same, thus the IEP may provide an alternative biologically based measure (going beyond goodness of fit type) of the adequacy of the chosen maximum entropy model.

Unfortunately, we do not yet know how to quantify the spiking activity in ways that yield the most meaningful insights into the relationship between the activity patterns and nervous system function and we are still looking for better conceptual-mathematical frameworks to better describe and understand spiking dynamics. We believe IEP may play an important conceptual role in future studies as help thinking about this dynamics.

However, there are two main drawbacks of our approach, both inherited from the MEP. The first is that the MEP assumes stationarity in the data, which is not a common situation from recordings in neuronal systems, so requires careful experimental control to approach this condition. The second is methodological and related to the fact that the Markov transition matrix as presented here is obtained from the transfer matrix technique, so it may require an important computational effort for large-scale and long memory spiking neuronal networks. Indeed as discussed in [18] this approach can reliably recover Markov transition matrices for systems of *N* neurons and memory R−1 that satisfies N×R≤20. However, new methods based on Monte Carlo methods can overcome this limitation [46].

There is a lot of room for progress going beyond the scope of this work, one possibility is to quantify the IEP for different choices of non-synchronous constraints and binning sizes on biological spike train recordings. A more ambitious goal would be to link the IEP as a signature of an underlying physiological process depending on time such as adaptation or learning. IEP is a much broader concept which can also be measured along non-stationary trajectories, thus IEP can be measured for time-dependent models where transition probabilities are explicitly given or can be computed (for example the GLM [8]). Previous studies in the context of spike train statistics have measured the dynamical entropy production in spiking neuron networks using a deterministic approach based on the Pesin identity (sum of positive Lyapunov exponents) [47]. There are relationships between the deterministic and stochastic dynamics [48], and some interpretations of deterministic dynamical entropy production with information loss which should be investigated in more detail, in particular, if these relationships bring new knowledge in the field of computational neuroscience.

We have focused on spike train statistics, but our results are not restricted to this field and can be applied wherever Markov maximum entropy measures under constraints have to be inferred from data, especially for irreversible Markov chains arising from stochastic network theory [49], information theory [37], and finance [38], among other disciplines.

## Abbreviations

The following abbreviations are used in this manuscript:

MEP	Maximum entropy principle
MEMC	Maximum entropy Markov chain
IEP	Information entropy production
KSE	Kolmogorov–Sinai entropy
IF	Integrate-and-Fire
GLM	Generalized Linear model
NESS	Non-equilibrium steady states

## Symbol List

$\sigma_{k}^{n}$	Spiking state of neuron *k* at time *n*.
$\sigma^{n}$	Spike pattern at time *n*
$\sigma^{n_{1},n_{2}}$	Spike block from time $n_{1}$ to $n_{2}$.
$A_{T}\left(f\right)$	Empirical Average value of the observable *f* considering *T* spike patterns.
$\Sigma_{N}^{L}$	Set of spike blocks of *N* neurons and length *L*.
S[μ]	Entropy of the probability measure μ.
H	Potential function.
P[H]	Free energy or topological pressure.
